# Long-Term Protective Effect of Serial Infections with H5N8 Highly Pathogenic Avian Influenza Virus in Wild Ducks

**DOI:** 10.1128/jvi.01233-22

**Published:** 2022-09-13

**Authors:** Valentina Caliendo, Lonneke Leijten, Marco W. G. van de Bildt, Marjolein J. Poen, Adinda Kok, Theo Bestebroer, Mathilde Richard, Ron A. M. Fouchier, Thijs Kuiken

**Affiliations:** a Department of Viroscience, Erasmus University Medical Center, Rotterdam, The Netherlands; Peter Doherty Institute for Infection and Immunity

**Keywords:** HPAIV, H5N8, mallards, tufted ducks

## Abstract

Highly pathogenic avian influenza viruses (HPAIVs) of the Goose/Guangdong (Gs/Gd) lineage are an emerging threat to wild birds. In the 2016–2017 H5N8 outbreak, unexplained variability was observed in susceptible species, with some reports of infected birds dying in high numbers and other reports of apparently subclinical infections. This experimental study was devised to test the hypothesis that previous infection with a less-virulent HPAIV (i.e., 2014 H5N8) provides long-term immunity against subsequent infection with a more-virulent HPAIV (i.e., 2016 H5N8). Therefore, two species of wild ducks—the more-susceptible tufted duck (Aythya fuligula) and the more-resistant mallard (Anas platyrhynchos)—were serially inoculated, first with 2014 H5N8 and after 9 months with 2016 H5N8. For both species, a control group of birds was first sham inoculated and after 9 months inoculated with 2016 H5N8. Subsequent infection with the more-virulent 2016 H5N8 caused no clinical signs in tufted ducks that had previously been infected with 2014 H5N8 (*n* = 6) but caused one death in tufted ducks that had been sham inoculated (*n* = 7). In mallards, 2016 H5N8 infection caused significant body weight loss in previously sham-inoculated birds (*n* = 8) but not in previously infected birds (*n* = 7).

**IMPORTANCE** This study showed that ducks infected with a less-virulent HPAIV developed immunity that was protective against a subsequent infection with a more-virulent HPAIV 9 months later. Following 2014 H5N8 infection, the proportion of birds with detectable influenza nucleoprotein antibody declined from 100% (8/8) in tufted ducks and 78% (7/9) in mallards after 1 month to 33% (2/6) in tufted ducks and 29% (2/7) in mallards after 9 months. This finding helps predict the expected impact that an HPAIV outbreak may have on wild bird populations, depending on whether they are immunologically naive or have survived previous infection with HPAIV.

## INTRODUCTION

Highly pathogenic avian influenza viruses (HPAIVs) of the Goose/Guangdong (Gs/Gd) lineage are an emerging threat to wild birds (1–11). Since the emergence of the H5 A/goose/Guangdong/1/96 lineage in 1996, HPAIVs have successfully adapted and circulated widely in several wild bird species (3, 8). Wild waterfowl now constitute an important vector for HPAIVs and their global spread. Wild *Anseriformes* offer HPAIVs the great evolutionary advantage to travel via their migratory routes and the opportunity to change their genetical pool by reassorting with circulating low-pathogenicity avian influenza viruses (LPAIVs) (7, 12).

Examples of these successful mechanisms are the numerous global incursions of the subtype H5N8, clade 2.3.4.4, in the 2014–2015, 2016–2017, and 2020–2021 seasons, which to date is responsible for the highest number of HPAIV outbreaks in wild birds. During the H5N8 outbreak in 2014–2015, the virus spread long distances from Asia to Europe and North America via infected migratory birds (1, 2, 6). Epidemiological analysis and experimental infection studies showed that wild ducks (including Eurasian wigeons, Anas penelope, and mallards, Anas platyrhynchos) can be infected with 2014 H5N8 virus without clinical or pathological evidence of disease (13–20). Two years later, the 2016–2017 H5N8 outbreak also spread intercontinentally along the wild bird migratory pathways and caused a large and widespread highly pathogenic avian influenza (HPAI) epidemic in Europe. In the Netherlands alone, more than 13,600 wild birds were reported dead, and up to 5% of the wintering populations of tufted ducks (Aythya fuligula) and Eurasian wigeons (more than 2,500 birds for each species) may have died (9). The 2020–2021 outbreak also caused extensive mortality in wild birds and for the first time in geese. In the Netherlands, Barnacle geese (*Branta leucopsis*) were the most affected species (1, 2). During these outbreaks, HPAIV H5N8 was isolated from apparently clinically healthy free-living wild ducks (mainly Eurasian wigeons and mallards), with some birds also presenting HPAI H5 virus-specific antibodies (7, 16, 17).

It is not known why there were so many differences in outcome within a single species in the 2016–2017 outbreak; in particular for the Eurasian wigeon, there were both events with HPAI-related high mortality as well as events of live, HPAIV-positive, but otherwise apparently healthy birds (9, 16). In the field, the fact that apparently healthy birds have serum antibodies against avian influenza viruses (AIVs) is an indication that birds can survive HPAIV infections. However, it is not understood what determines that some wild ducks die from infection but others do not. Experimental studies comparing the pathogenesis of infection with 2014 H5N8 versus 2016 H5N8 showed that 2016 H5N8 had an augmented virulence for two duck species (13, 18). Experimental studies have shown the effect of short-term protection after serial HPAIV infection in Pekin ducks (Anas platyrhynchos *domesticus*) and mallards (19). However, it is not known whether previously infected birds can survive subsequent challenges after long intervals, for example, between two consecutive autumn migrations. It is also not known whether this is valid for all bird species or that there are differences in outcome between species that are highly susceptible to disease (e.g., tufted duck) and less-susceptible species (e.g., mallard).

This experimental study was devised to complement field observations and to provide an explanation for the dynamics of the infection survival rate during the 2014–2015 and 2016–2017 H5N8 outbreaks in wild birds. The hypothesis was that a previous infection with a less-virulent HPAIV (i.e., 2014 H5N8) provides a long-term immunity against a subsequent infection with a more-virulent HPAIV (i.e., 2016 H5N8). To test this hypothesis, two groups of wild ducks (either mallards or tufted ducks) were serially inoculated, first with 2014 H5N8 and after 9 months with 2016 H5N8. For both species, a control group of birds was first sham inoculated and after 9 months inoculated with 2016 H5N8. We used species that differed in their susceptibility to disease from HPAIV infection, with the mallard being less susceptible and the tufted duck being more susceptible. We had planned to include the Eurasian wigeon as another less-susceptible species, but we could not source sufficient birds. The timing of the inoculations was planned to correspond with the autumn peak of AIV infections, to better mimic the field dynamics of the outbreaks. We hypothesized that all mallards, both sham inoculated and 2014 H5N8-inoculated, and possibly 2014 H5N8-inoculated tufted ducks would survive the infection with 2016 H5N8. Conversely, we hypothesized that the sham-inoculated tufted ducks would not survive the infection with 2016 H5N8, because they lacked protective immunity. The sera of the inoculated ducks were tested with a hemagglutination inhibition (HI) assay against the two HPAIVs used in the experiment and against a more recent HPAIV from clade 2.3.4.4b to assess the breadth of the immune response of the birds.

## RESULTS

### Inoculation with HPAIV 2014 H5N8 (first inoculation).

**FIG 1:**
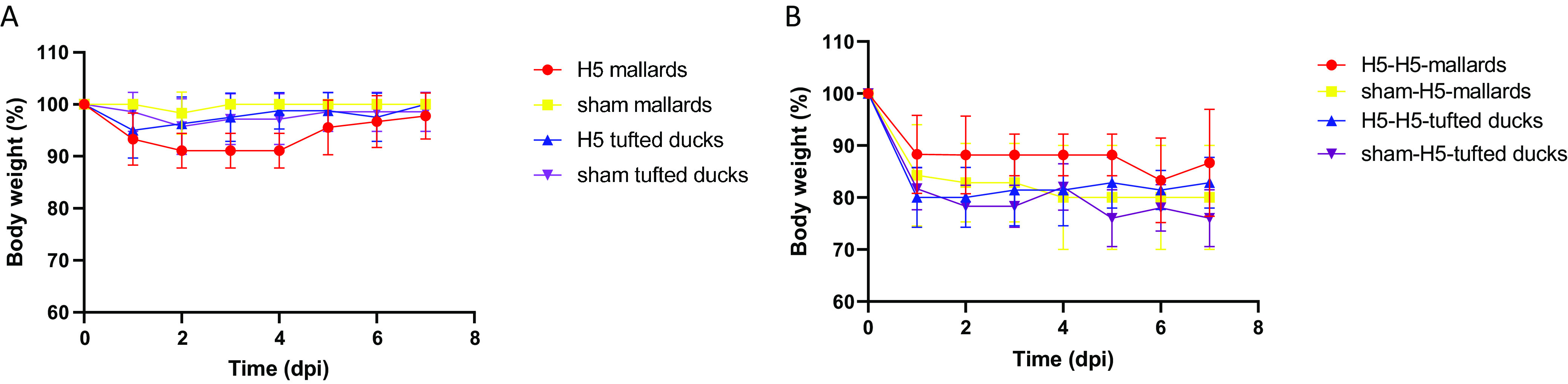
Body weight loss after inoculation with HPAIV 2014 H5N8 or sham inoculation (A) and after inoculation with HPAI 2016 H5N8 (B) in mallards and tufted ducks. After inoculation, ducks were weighed and means and standard deviations of the relative weight loss (compared to the body weight on the day of inoculation) were calculated.

**FIG 2:**
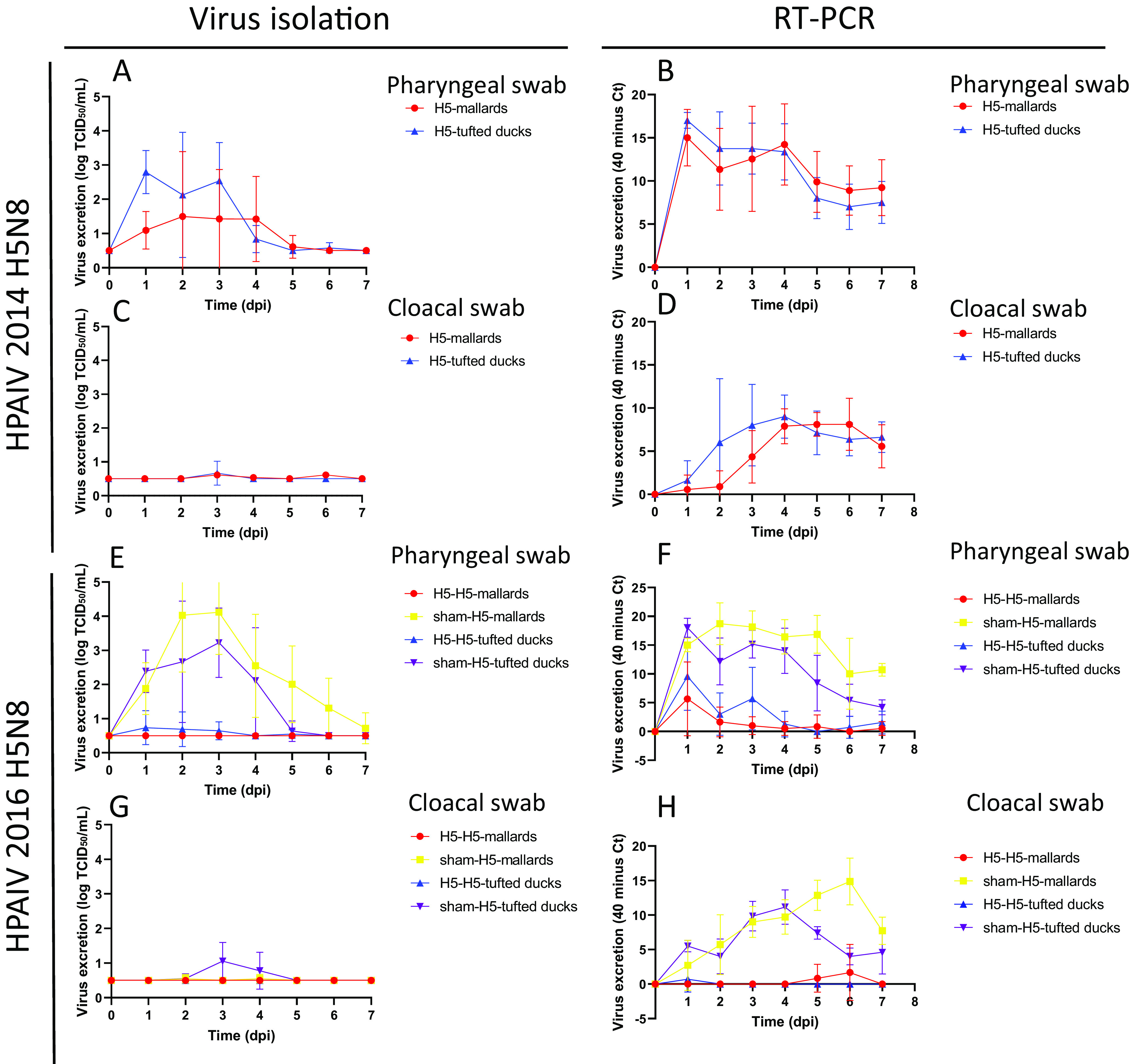
Virus excretion in mallards and tufted ducks of. (A and B) Excretion of HPAIV 2014 H5N8 from the pharynx, based on virus isolation (A) and virus detection by RT-PCR (B). (C and D) Excretion of HPAIV 2014 H5N8 from the cloaca, based on virus isolation (C) and virus detection by RT-PCR (D). Excretion of HPAIV 2016 H5N8 from the pharynx, based on virus isolation (E) and virus detection by RT-PCR (F). (G and H) Excretion of HPAIV 2014 H5N8 from the cloaca, based on virus isolation (G) and virus detection by RT-PCR (H). Symbols indicate mean values and error bars indicate standard errors.

**FIG 3:**
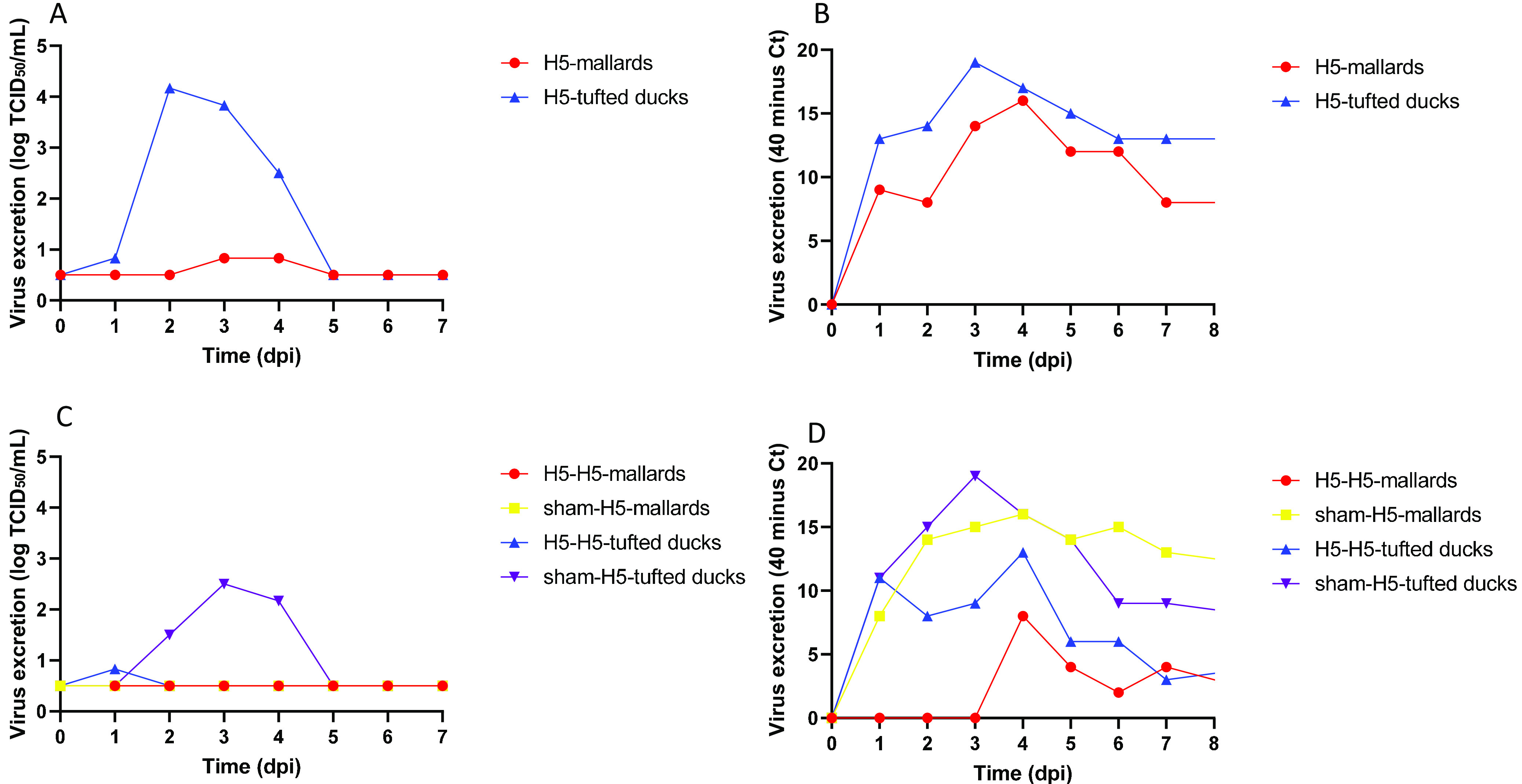
Virus contamination of drinking water for mallards and tufted ducks. (A and B) Results after inoculation with HPAIV 2014 H5N8, based on virus isolation (A) and virus detection by RT-PCR (B). (C and D) Results after inoculation with HPAIV 2016 H5N8, based on virus isolation (C) and virus detection by RT-PCR (D).

At the first inoculation, all 17 inoculated birds became infected, 100% (17/17) based on reverse transcription-PCR (RT-PCR) and 94% (16/17) based on virus isolation (Table 1). There was no significant loss of body weight of the infected tufted ducks (“H5-tufted ducks”) compared to the control tufted ducks (“sham-tufted ducks”). One H5-tufted duck presented with transient, excessive eye blinking at 24 h postinoculation (p.i.). There was significant loss of body weight (paired *t* test, *P* < 0.002) of the infected mallards (“H5-mallards”) compared to the control mallards (“sham-mallards”) (Fig. 1A). Pharyngeal and cloacal excretion levels of infectious 2014 H5N8 between H5-mallards and H5-tufted ducks were not statistically different (Kruskal-Wallis test, area under the curve [AUC] for excretion from 0 to 7 days p.i.) (Table 2). For both groups, pharyngeal excretion exceeded cloacal excretion according to virus isolation and RT-PCR (paired *t* test, *P* < 0.001) (Fig. 2A to D). Virus concentration in drinking water samples of the H5-tufted ducks exceeded that of the H5-mallards, according to virus isolation (*t* test, *P* < 0.02) but not according to RT-PCR (Fig. 3A and B). However, the different drinking behaviors that the two species manifested during the experiment (mallards generally used more water than tufted ducks, both for drinking and preening their feathers) may have affected this evaluation.

**TABLE 1:** Health status and virus excretion of 32 ducks experimentally inoculated with HPAIV H5N8 (2014 H5N8 and 2016 H5N8)

Common name (taxonomic name)	Group	Virus	No. of birds	No. of birds with clinical signs	No. of birds excreting from pharynx based on:		No. of birds excreting from cloaca based on:	
					Virus isolation	PCR	Virus isolation	PCR
Mallard (Anas platyrhynchos)	H5-mallards	H5N8 2014	9	0	8	9	3	9
Mallard (Anas platyrhynchos)	H5-H5-mallards	H5N8 2016	6	0	0	3	0	2
Tufted duck (*Aythya fuligula*)	H5-tufted ducks	H5N8 2014	8	8	8	8	2	8
Tufted duck (*Aythya fuligula*)	H5-H5-tufted ducks	H5N8 2016	7	0	2	6	0	2
Mallard (Anas platyrhynchos)	Sham-mallards	Sham	8	0	NP^*a*^	NP	NP	NP
Mallard (Anas platyrhynchos)	Sham-H5-mallards	H5N8 2016	7	0	7	7	2	7
Tufted duck *(Aythya fuligula*)	Sham-tufted ducks	Sham	7	0	NP	NP	NP	NP
Tufted duck (*Aythya fuligula*)	Sham-H5-tufted ducks	H5N8 2016	6	6	6	6	4	6

a NP, not performed.

**TABLE 2:** Level and duration of virus excretion of HPAIVs 2014 H5N8 and 2016 H5N8 from the pharynx and cloaca in mallards and tufted ducks^*a*^

Host	Virus	Pharyngeal swabs			Cloacal swabs			Water		
		AUC (mean ± SE)	Median (dpi)	Peak (dpi)	AUC (mean ± SE)	Median (dpi)	Peak (dpi)	AUC (mean ± SE)	Median (dpi)	Peak (dpi)
H5-mallards	H5N8 2014	7 ± 1.9	4.5	2	3.7 ± 0.2	4	3	4.1 ± 0	4.5	3
H5-H5-mallards	H5N8 2016	3.5 ± 0	0	0	3.5 ± 0.1	0	2	3 ± 0	0	1
H5-tufted ducks	H5N8 2014	4.1 ± 0.5	4	1	3.6 ± 0.2	0	3	12.8 ± 0	4	2
H5-H5-tufted ducks	H5N8 2016	4.1 ± 2.1	4	1	3.5 ± 0	0	0	3.8 ± 0	1.5	1
Sham-H5-mallards	H5N8 2016	16 ± 2.1	5	3	3.5 ± 0.1	5	2	3.5 ± 0	0	0
Sham-H5-tufted ducks	H5N8 2016	12 ± 1.8	4	3	4.3 ± 0.5	4.5	3	8.1 ± 0	4	3

a Data are based on virus isolation from ducks and also virus contamination of drinking water. AUC, area under the curve, summarizes infectious virus excretion from day 0 to 7 postinoculation. dpi, days postinoculation. The minimal detection limit of virus isolation was log 0.5 TCID50/mL, and the minimal area under the curve from day 0 to 7 postinoculation was 3.

**FIG 4:**
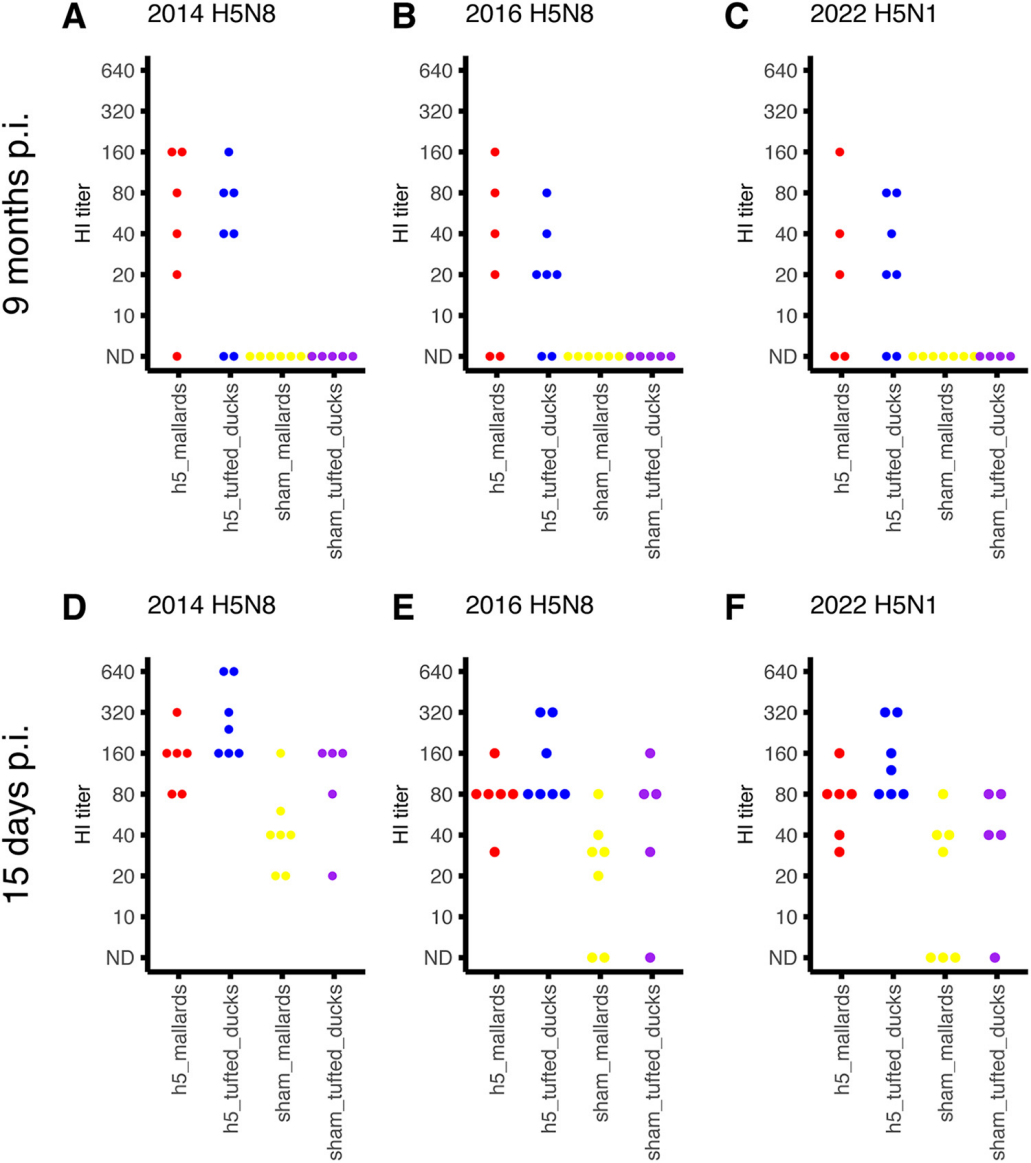
Breadth of immune response. Sera from the inoculated ducks obtained 9 months after the first inoculation (A to C) and 15 days after the second inoculation (D and F) were tested with the HI test against 2014 H5N8 (A and D), 2016 H5N8 (B and E), or 2022 H5N1 A/Caspian-gull/Netherlands/1/2022 (C and F). The HI titers of individual animals are depicted on the *y* axis. ND, not detected.

At 1 month p.i., 87% (7/8) H5-mallards and 100% H5-tufted ducks had detectable H5-specific and nucleoprotein (NP)-specific antibodies (Table 3). Just before the second inoculation, at 9 months p.i., these percentages had decreased to 83% (5/6) H5-specific and 33% (2/6) NP-specific antibodies for H5-mallards and 71% (5/7) H5-specific and 28% (2/7) NP-specific antibodies for H5-tufted ducks. Serum antibodies of the H5-mallards and H5-tufted ducks cross-reacted against the 2016 H5N8 virus in the hemagglutination inhibition (HI) test (Fig. 4).

**TABLE 3:** Number of birds with detectable serum antibodies after inoculation with HPAIV 2014 or 2016 H5N8

Time p.i. and strain	No. of birds with antibodies/total no. inoculated^*a*^			
	Mallards		Tufted ducks	
	NP	HI	NP	HI
1 mo (2014 H5N8)	7/9	7/9	8/8	7/7
2 mo (2014 H5N8)	6/9	7/9	7/7	7/7
3 mo (2014 H5N8)	1/9	6/9	3/7	5/7
5 mo (2014 H5N8)	2/7	5/7	2/7	4/7
6 mo (2014 H5N8)	2/7	6/7	2/7	7/7
7 mo (2014 H5N8)	2/6	5/6	2/7	6/7
8 mo (2014 H5N8)	2/6	4/6	2/7	7/7
9 mo (2014 H5N8)	2/6	5/6	2/7	5/7
15 days (2016 H5N8)	13/13	13/13	12/12	12/12

a Ducks were tested for antibodies to NP via ELISA and for 2014 H5N8-specific antibody via hemagglutination inhibition (HI) test.

### Inoculation with HPAIV 2016 H5N8 (second inoculation).

After the second inoculation, 100% (13/13) of control birds (seven sham-H5 mallards and six sham-H5 tufted ducks) became infected, based on both RT-PCR and virus isolation; 50% (3/6) of the H5-H5 mallards became infected based on RT-PCR and 33% (2/6) were infected based on virus isolation; 85% (6/7) of the H5-H5 tufted ducks became infected based on RT-PCR and 28% (2/7) were infected based on virus isolation (Table 1).

**FIG 5:**
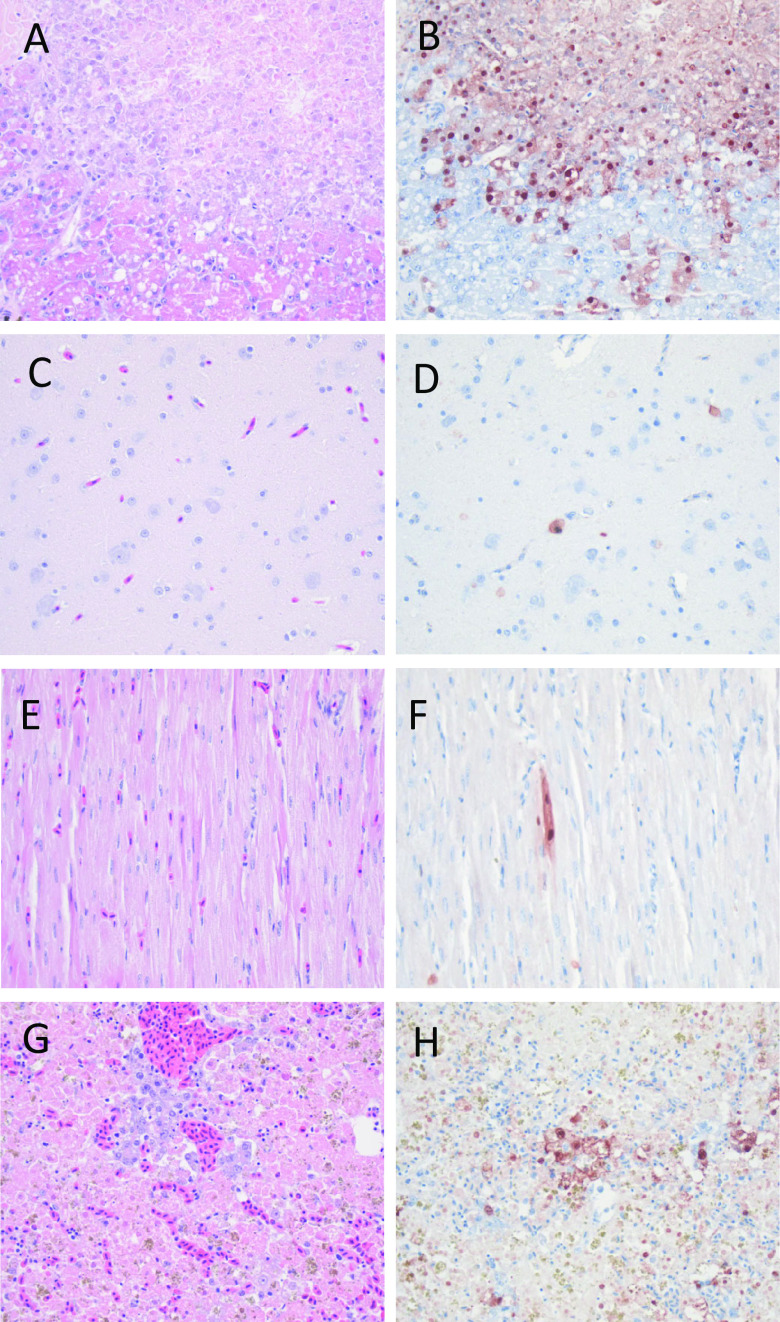
Tissue sections stained by hematoxylin and eosin (left) or by immunohistochemistry (right) from a sham-H5-tufted duck found dead at 4 days p.i. with HPAIV 2016 H5N8. The pancreas showed severe necrosis (A) and abundant expression of influenza antigen (B); the brain showed mild inflammation (C) and moderate expression of influenza antigen (D); the heart showed mild inflammation (E) and moderate expression of influenza antigen (F); and the liver showed necrotizing hepatitis (G) and abundant expression of influenza antigen (H).

There was no significant loss of body weight of H5-H5 tufted ducks compared to the sham-H5 tufted ducks (Fig. 1B). One of the sham-H5 tufted ducks presented with general weakness and died overnight at day 4 p.i. Postmortem examination macroscopically revealed multifocal areas of necrosis in the pancreas. Histologically, the necrotic areas colocalized with influenza virus antigen expression in pancreatic acinar cells (Fig. 5). The liver had histological evidence of necrotizing hepatitis with abundant virus antigen expression in hepatocytes. Mild virus antigen expression was also present in brain (neurons), heart (myocytes), and lungs and air sacs (epithelial cells). Virus antigen expression was not observed in the intestine. Virus was detected in tissues of all the main organs by virus isolation and RT-PCR, indicating that the virus had spread systemically (Table 4).

**TABLE 4:** Amounts of HPAIV 2016 H5N8 present in tissues of a sham-H5-tufted duck that died at day 4 p.i., based on RT-PCR and virus titration

Organ	RT-PCR^*a*^	Virus titration (log TCID_50_/mL)
Brain	19	5.1
Trachea	14	6.5
Air sac	18	4.8
Lung	12	5.5
Heart	12	5.1
Stomach	19	5.5
Jejunum	18	5.5
Colon	16	5.5
Pancreas	16	7.5
Liver	12	7.5
Spleen	16	5.5
Kidney	20	6.1

a RT-PCR results are cycle threshold values.

There was a significant loss of body weight (paired *t* test, *P* < 0.01) of sham-H5-mallards compared to the H5-H5-mallards, without any other clinical signs of disease. However, both groups of mallards, in particular H5-H5-mallards, had established a strong dominance hierarchy (dominant birds had priority access to food and water compared to subordinate birds) that may have interfered with body weight evaluation.

Pharyngeal excretion significantly differed between groups (one-way analysis of variance of AUC for 0 to 7 days p.i., *P* < 0.001) (Fig. 2). The mean quantity of virus excreted per group from 0 to 7 days p.i. was highest for sham-H5-mallards, followed by sham-H5-tufted ducks and H5-H5-tufted ducks, and was lowest for H5-H5-mallards, according to virus isolation and RT-PCR. Pharyngeal excretion was statistically higher for sham-H5-mallards versus H5-H5-mallards (*t* test, *P* < 0.04) and for sham-H5-tufted ducks versus H5-H5-tufted ducks (*t* test, *P* < 0.02). Pharyngeal excretion exceeded cloacal excretion in the different groups. Cloacal excretion was scarce, and there was no statistically significant difference in cloacal excretion between groups, according to virus isolation. Cloacal excretion was scarce for H5-H5-mallards and H5-H5-tufted ducks, and cloacal excretion was statistically higher for sham-H5-mallards versus H5-H5-mallards (*t* test, *P* < 0.0001) and for sham-H5-tufted ducks versus H5-H5-tufted ducks (*t* test, *P* < 0.0001), according to RT-PCR. Concentration of virus in drinking water was scarce for the different groups except that for sham-H5-tufted ducks, according to virus titration; virus concentration in the water was statistically higher for sham-H5-tufted ducks versus H5-H5-tufted ducks (*t* test, *P* < 0.04), according to the RT-PCR.

Fifteen days after the second inoculation, all the birds had detectable serum NP-specific and H5-specific antibodies against the 2014 and 2016 H5N8 strains (Fig. 4A and B). Serum antibody cross-reaction was detected consistently only against the 2022 H5N1 (Fig. 4C). All survivor birds were euthanized at the end of the experiment. Macroscopic and histologic examination of their organs did not show any abnormal findings, and thus no further tests were performed.

## DISCUSSION

This study showed that ducks infected with a less-virulent HPAIV (2014 H5N8) developed a long-term immunity that was protective against a subsequent infection with a more-virulent HPAIV (2016 H5N8). This finding is consistent with the previously demonstrated long-term protective effect of LPAIV infection and short-term protective effect of HPAIV infection (19, 20) and further demonstrates that long-term protection applies to HPAIV reinfections in relevant wild duck species.

Prior infection with the less-virulent 2014 H5N8 protected against clinical signs from the more-virulent 2016 H5N8 infection nearly 1 year later. After inoculation with 2014 H5N8, both mallards and tufted ducks became infected and excreted infectious virus. In accordance with our hypothesis, all mallards, both sham inoculated and 2014-H5N8 inoculated, as well as 2014-H5N8-inoculated tufted ducks, survived the infection with 2016 H5N8. The 2014-H5N8-infected mallards showed detectable clinical signs of disease (i.e., weight loss), and one of the 2014-H5N8-infected tufted ducks showed clinical signs of disease consisting of mild neurological signs. After inoculation with the more-virulent 2016 H5N8, control mallards showed weight loss and control tufted ducks manifested increased mortality, although less than we had hypothesized, with only a 14% (1/7) mortality rate. This incongruence could be due to the fact that birds in our study were all in good health preinfection and may have had a greater chance of survival compared to free-range wild birds (12).

Pharyngeal viral excretion was higher than cloacal excretion, in accordance with previous experimental studies in these and other wild bird species (13–15). Infectious virus was excreted up to 5 days p.i. and was likely transferred from the pharynx to drinking water. This fits with the idea that water can be an important source of infection of HPAIV for birds (13).

Antibody responses in ducks are still only partially understood (21). Studies on gulls and ducks have shown that serum antibody levels do not correlate with protection against LPAIV infection (20, 22). In our study, we screened for both H5-specific antibodies (by HI test) and NP-specific antibodies (by enzyme-linked immunosorbent assay [ELISA]). The HI assay is historically considered to be the reference test for serological surveillance (23). Nowadays, NP ELISAs are more often used to assess the serological response against AIVs in multiple bird species, and they frequently replace the HI test for research and diagnostic purposes in wild birds and poultry (24–28). After inoculation with 2014 H5N8, NP-specific antibodies were detected in a limited number of birds for a limited period of time, and their presence generally was not associated with previous infection, nor with reinfection outcome. Overall, a higher percentage of birds tested positive for H5-specific antibodies than for NP-specific antibodies throughout the experiment. Sera testing positive solely with the HI test were observed around 4 to 9 months p.i. with the 2014 H5N8 virus. This observation might have been the consequence of a difference in sensitivity between the assays, or of a difference in the kinetics between NP-specific and H5-specific antibodies (23). One month postinoculation with 2014 H5N8, 88% of ducks had detectable NP-specific antibodies; however, this percentage was much lower at 9 months p.i., when only 33% of inoculated mallards and 28% of infected tufted ducks presented serum antibodies against avian influenza virus. After inoculation with 2016 H5N8, there was no difference in survival between previously infected birds with or without detectable levels of H5-specific and NP-specific antibodies. This result is in line with the experimental study of Verhagen et al. 2015 (20) and further demonstrates that it is difficult to assess the level of protection against disease of an individual bird based on serological tests only. Furthermore, the value of serological tests in wild birds is limited in time; in particular, NP-specific antibodies have a shorter window of detection than H5-specific antibodies.

Mallards were our model for less susceptible species and a comparison group for elaborating on the dynamics of the 2016 H5N8 outbreak in the Eurasian wigeon population. All mallards survived the two infections, and control mallards excreted more virus postinfection than control tufted ducks. The mallard excretion pattern of 2014 H5N8 was similar to those described by Keawcharoen et al. (in 2008) for 2005 H5N1 and by van den Brand et al. (in 2018) for 2014 H5N8 (14, 15). In concurrence with those studies, this study found that control mallards excreted a relatively high quantity of infectious virus and thus may be suitable vectors of HPAI H5 viruses. However, after reinfection with HPAIV H5N8, mallards did not excrete any infectious virus. This result may have an important consequence in the field, because it excludes a significant role for previously infected mallards (and possibly other duck species) in spreading infectious, antigenically similar HPAIVs. Given the increasing frequency of new HPAIV outbreaks and the long-term protective effect of previous HPAIV infections, older mallards may not contribute to the persistence of HPAIV in the bird population. This also implies that shorter-lived wild waterbird species, thus with a larger proportion of juvenile birds in the population, may be more important as reservoirs of HPAIV than longer-lived birds. Wild mallards are relatively short-lived (around 1to 3 years) on account of heavy hunting pressure, while Eurasian wigeons and tufted ducks are relatively longer-lived species (around 3 and 10 years, respectively) (29–31).

Tufted ducks were our model for more susceptible species, based on both field and experimental data. Unusually high mortality of tufted ducks was reported during the 2016 H5N8 outbreak, as well as the earlier 2005–2006 H5N1 outbreak (9, 11). Experimental studies showed that tufted ducks, and diving ducks more generally, develop fatal disease after infection with HPAIV H5N1 (15); however, infection with 2014 H5N8 in common pochards (Aythya ferina, a diving duck species) was asymptomatic (14). Our study showed that, under experimental conditions, previous infection protected tufted ducks 100% from clinical signs, including body weight loss and mortality, compared to sham-inoculated tufted ducks that were clinically affected. This could explain why susceptible species (e.g., tufted ducks and Eurasian wigeons) that are infected in repeated HPAIV outbreaks have disparate outcomes: some remain apparently healthy because they have been infected in previous years, while others die because they are immunologically naive.

Based on this observation, we could predict the impact of future HPAIV outbreaks on wild bird populations: previously infected bird populations (i.e., survived infection with a less-virulent HPAIV) will have higher chances of surviving a subsequent infection with a more-virulent HPAIV in the following influenza outbreak. The effect of this implication is suggested in the trend of the 2020–2021 HPAIV H5 outbreak. During this outbreak, high mortality in duck populations was not reported, which could be related to the long-term protective effect of their exposure to previous HPAIVs (e.g., 2016 H5N8). Conversely, goose species, like the Barnacle goose (*Branta leucopsis*), that were not reported infected during previous HPAIV outbreaks had an unusually high number of deaths related to the infection in 2020–2021 (1, 2), possibly because they lacked protective immunity from previous exposures. However, we cannot exclude the possibility that geese are inherently more susceptible to severe disease with 2020 H5 viruses.

Experimental and field studies showed that prior infections with both homologous and heterologous AIV can prevent reinfection, reduce the duration and extent of AIV shedding, or result in a higher infective dose being required for subsequent infections (19, 20, 32, 33). In this study, protection was confirmed because the 2014 H5N8 infected ducks presented cross-reactive antibodies against the antigenically similar 2016 H5N8 virus and against the more recent 2022 H5N1. Pre-exposure to LPAIVs can also provide some level of protective immunity against a subsequent HPAIV infection (19, 33). We would expect, however, that exposure to HPAIV H5 exposure provides a stronger protective effect than to a LPAIV H5 and that exposure to other LPAIV subtypes provides an even lower protective effect.

In conclusion, this study showed that ducks infected with a less-virulent HPAIV developed a long-term immunity that was protective against a subsequent infection with a more-virulent HPAIV nearly 1 year later. This finding will help in understanding and potentially predicting the expected impact that an HPAIV outbreak may have on bird populations, depending on whether they are previously exposed or naive to HPAIV infections. This study also showed that serum antibodies post-HPAIV infection detected by NP ELISA have a short window of detection, which should be taken in account during surveillance and assessment of outbreaks.

## MATERIALS AND METHODS

### Virus preparation.

The two HPAIVs used in this study for the serial inoculation of the wild ducks were 2014 H5N8 (A/Eurasian Wigeon/NL/EMC-1/2014_2.3.4.4c), isolated from the feces of a nonsymptomatic wild Eurasian wigeon, and 2016 H5N8 (A/Eurasian Wigeon/NL/4/2016_2.3.4.4b), isolated from a dead wild Eurasian wigeon. Full-length hemagglutinin (HA) and neuraminidase (NA) sequences and full genome sequences for these two virus isolates were obtained by Sanger sequencing, and sequences were deposited in a public database (https://gisaid.org/; EPI_ISL_168746 and EPI_ISL_255912). The viruses were propagated by two passages in Madin-Derby canine kidney (MDCK) cells. The harvested supernatant had a titer of 1 × 10^7^ median tissue culture infectious dose (TCID_50_)/mL and was diluted with phosphate-buffered saline (PBS) to 1 × 10^6^ TCID_50_/0.1 mL. These viruses were chosen because they both circulated in wild birds during the correspondent outbreaks and were expected to reproduce the field infections more realistically. All experiments with HPAIVs (2014 H5N8 and 2016 H5N8) were performed under biosafety level 3 (BSL3) conditions.

To test the breadth of the immune response of the inoculated wild ducks, the sera of the birds were tested with an HI test for antibody cross-reactivity against previously and more recently circulating H5 viral strains, namely, 2022 H5N1 (A/Caspian-gull/Netherlands/1/2022, clade 2.3.4.4b), A/Mallard/Sweden/49/2002 (non A/goose/Guangdong/1/1996 H5), A/muscovy-duck/Vietnam/NCVD-KA426/2013 (clade 1.1.2), A/duck/GizA/15292S/2015 (clade 2.2.1.2), A/Nepal/19FL1997/2019 (clade 2.3.2.1a), A/duck/Vietnam/NCVD-1584/2012 (clade 2.3.2.1e), and A/Guangdong/18SF020/2018 (clade 2.3.4.4h). These antigens were available in-house at the Viroscience Department of the Erasmus Medical Center as recombinant virus stocks propagated in MDCK cells, which contained the H5 HA of the respective viral strain without a multibasic cleavage site, in the background of seven gene segments of A/Puerto Rico/8/1934.

### Animals.

Two species of ducks were inoculated experimentally: one species of diving duck (tufted duck, *Aythya fuligula*) and one species of dabbling duck (mallard, Anas platyrhynchos). All ducks used for the infection experiments were captive bred. Birds were 4 to 5 months of age at the time of first inoculation. Blood samples, cloacal swabs, and pharyngeal swabs were collected from all ducks 1 week before inoculation. Sera were analyzed, as described below, by using a commercially available influenza A virus antibody ELISA kit for the detection of antibodies against nucleoprotein (Idexx) according to the manufacturer’s instructions (16). Swabs were tested by RT-PCR (14, 15). Prior to inoculation, no duck had antinucleoprotein antibody, and all tested negative by RT-PCR targeting the matrix gene.

Ducks were first inoculated with 2014 H5N8 and then after 9 months with 2016 H5N8. For both species, a control group of birds was first sham inoculated and then after 9 months inoculated with 2016 H5N8. The time between the two inoculations was chosen to approximate the time period between two consecutive autumns, when infection usually takes place in the field in Europe. Drinking water was sampled after the inoculations to check if birds could infect each other by contact with water.

### Experimental design.

For the infection with 2014 H5N8 (first inoculation), nine mallards and eight tufted ducks were housed in two negatively pressurized isolator units (Table 1). The method of inoculation was a standard method established by the Delta-Flu Consortium. Each bird in these two groups (infected group, H5-mallards and H5-tufted ducks) was inoculated intrachoanally with 1 × 10^6^ TCID_50_ HPAIV 2014 H5N8 in 0.1 mL. At the same time, eight mallards and seven tufted ducks were sham inoculated intrachoanally with 0.1 mL of PBS (naive group, sham-mallards and sham-tufted ducks). Each day, a qualified veterinarian assessed all birds for clinical signs of disease. Body weights, water samples, and cloacal and pharyngeal swabs were collected daily for the first 7 days and every 2 days thereafter. After inoculation, ducks were weighed and means and standard deviations for relative weight loss (compared to the body weight at the day of inoculation) were calculated.

Pharyngeal and cloacal swabs were collected using sterile cotton swabs, and each swab was placed in 1 mL virus transport medium (14, 15). Each drinking bucket held a volume of 5 liters, and water in the container was replaced daily. From each drinking bucket just before replacing water, 1 mL of water was collected in a sterile 2-mL tube containing 1 mL of virus transport medium. In order to test for the presence of infectious virus, to ensure that the birds would not carry infectious virus with them upon transfer to the BSL2 enclosure (after approval from the Erasmus University Biosafety Committee and in adherence to the Biosafety in Microbiological and Biomedical Laboratories-BMBL, 4th ed.), on days 9, 11, and 13 p.i. we collected swab samples from the feathers and feet of the birds, in addition to collecting cloacal and pharyngeal swabs. All the swabs collected on days 9, 11, and 13 p.i. tested negative for virus at virus isolation. On day 15 p.i., after it was confirmed that the infected group had stopped shedding infectious virus, the birds were moved to an indoor BSL2 enclosure. All ducks were clinically inspected monthly. During inspection, blood samples were collected to monitor the presence of antinucleoprotein antibody, and cloacal and pharyngeal swabs were collected to monitor virus excretion. All ducks tested negative for virus during monthly checks.

During the time between the two inoculations, three mallards and one tufted duck from the H5-infected groups and one mallard from the sham-inoculated group died from causes unrelated to HPAIV infection (trauma, aspergillosis, egg bound).

Nine months after infection with 2014 H5N8, six mallards and seven tufted ducks from the infected group and seven mallards and six tufted ducks from the naive group were inoculated intrachoanally with 1 × 10^6^ TCID_50_ HPAIV 2016 H5N8 in 0.1 mL (second inoculation). The birds were monitored and sampled using the established methods. On day 15 p.i., all surviving birds were euthanized.

### RT-PCR and virus titrations.

RNA isolation and RT-PCR were performed as described previously (14, 15). Briefly, RNA from swabs and tissue suspensions was isolated by using a MagNaPure LC system with the MagNaPure LC total nucleic acid isolation kit (Roche Diagnostics, Almere, the Netherlands). Real-time RT-PCR assays were performed on an ABI Prism 7500 sequence detection system (Applied Biosystems, Foster City, CA, USA) by using the TaqMan EZ RT-PCR core reagents kit (Applied Biosystems, Nieuwerkerk a/d IJssel, the Netherlands) according to the manufacturer’s instructions. For each run, the samples were prepared and processed in parallel with several negative and positive control samples. Virus titers were determined by serial 10-fold dilution of the homogenized tissue samples and swabs on MDCK cells, as described elsewhere (14, 15). Virus titrations were performed in triplicate.

### Antibody detection assays.

After the first inoculation, blood samples were taken monthly and tested for serum antibodies. Serum samples were tested for the presence of H5-specific and NP-specific antibodies. H5-specific antibodies were detected by using an HI test performed as described previously, using in-house turkey erythrocytes (34). Briefly, serum samples were treated overnight at 37°C with in-house-generated Vibrio cholerae filtrate containing receptor-destroying enzyme and subsequently inactivated at 56°C for 1 h and adsorbed with 10% turkey erythrocytes for 1 h at 4°C. Twofold serum dilutions of the serum samples were prepared in round-bottom 96-well plates, starting at a dilution of at 1:20; thus, the minimal detectable HI titer was 10. To each well containing 50 μL of serum dilution, 25 μL of PBS with 4 hemagglutinating units of virus was added to each well. Following incubation for 30 min at 37°C, 25 μL of 1% turkey erythrocytes was added to each well. Plates were incubated for 1 h at 4°C before reading the HI titer, which was determined as the reciprocal value of the highest serum dilution which completely inhibited erythrocyte agglutination.

NP-specific antibodies were detected by using a commercial blocking ELISA (bELISA; Idexx A Ab test; Idexx Laboratories BV, Hoofddorp, the Netherlands). Idexx bELISA is a high-throughput method for NP antibody quantitation in various host species (23–25, 28). The test uses mouse-derived monoclonal antibodies to compete with serum antibodies for binding to the antigen-labeled test kit and performs well in multiple avian species (24, 25). Applying the test per the manufacturer's recommendations results in good performance with 84% sensitivity and 100% specificity (35). Samples were tested according to the manufacturer’s instructions (16, 35). A sample was considered NP positive when the signal-to-noise ratio (i.e., ratio of the mean optical density [ODx] of the sample and ODx of the negative control) was 0.5 or lower.

### Pathologic examination and immunohistochemical testing.

Autopsies and tissue sampling were performed for all the birds, either those ill from HPAIV infection or those euthanized at the end of the experiment. After fixation of tissue samples in 10% neutral buffered formalin and embedding in paraffin, two sequential tissue sections were processed for histology with hematoxylin eosin staining or for immunohistochemistry with a monoclonal antibody against nucleoprotein of influenza A virus as the primary antibody for detection of influenza viral antigen (14, 15). The following tissues were examined: brain, trachea, lung, air sac, proventriculus, duodenum, pancreas, liver, jejunum, ileum, cecum, colon, spleen, kidney, heart, and adrenal gland.

### Ethics statement.

The study was approved by an independent animal experimentation ethical review committee and by the Dutch government (Stichting DEC consult) (permit number AVD1010020186744, protocol 18–6744-01).

### Data availability.

All data are available in the main text and in the GISAID database (http://platform.gisaid.org).
